# Comprehensive assessment of germline pathogenic variant detection in tumor-only sequencing

**DOI:** 10.1016/j.annonc.2022.01.006

**Published:** 2022-01-21

**Authors:** P. Terraf, F. Pareja, D. N. Brown, O. Ceyhan-Birsoy, M. Misyura, S. Rana, E. O’Reilly, M. I. Carlo, C. Aghajanian, Y. Liu, F. Derakhshan, G. Jayakumaran, B. Weigelt, M. Walsh, Z. Stadler, K. Offit, M. Ladanyi, M. Robson, A. Zehir, J. S. Reis-Filho, D. Mandelker

**Affiliations:** 1 Department of Pathology, Memorial Sloan Kettering Cancer Center, New York;; 2 Department of Medicine, Memorial Sloan Kettering Cancer Center, New York;; 3 Niehaus Center for Inherited Cancer Genomics, Memorial Sloan Kettering Cancer Center, New York, USA

**Keywords:** tumor-only sequencing, germline testing, precision medicine

## Abstract

**Background:**

Tumor-only sequencing, implemented for the identification of somatic variants, is oftentimes used for the detection of actionable germline variants. We sought to determine whether tumor-only sequencing assays are suitable for detection of actionable germline variants, given their importance for the delivery of targeted therapies and risk-reducing measures.

**Patients and methods:**

The detection of germline variants affecting moderate- and high-penetrance cancer susceptibility genes (CSGs) by tumor-only sequencing was compared to clinical germline testing in 21 333 cancer patients who underwent tumor and germline testing using the Food and Drug Administration (FDA)-authorized Memorial Sloan Kettering-Integrated Mutation Profiling of Actionable Targets (MSK-IMPACT) assay. Seven homologous recombination deficiency (HRD), two DNA damage response (DDR) and four mismatch repair (MMR) genes, as well as *NF1*, *RB1* and *TP53* were included in the analysis. FDA-authorized and New York State Department of Health-approved sequencing methods for germline, tumor/normal and tumor-only sequencing assays and analytical pipelines were employed.

****Results**:**

In patients who underwent tumor and germline sequencing, as compared to clinical genetic testing, tumor-only sequencing failed to detect 10.5% of clinically actionable pathogenic germline variants in CSGs, including 18.8%, 12.8% and 7.3% of germline variants in MMR, DDR and HRD genes, respectively. The sensitivity for detection of pathogenic germline variants by tumor-only sequencing was 89.5%. Whilst the vast majority of pathogenic germline exonic single-nucleotide variants (SNVs) and small indels were detected by tumor-only sequencing, large percentages of germline copy number variants, intronic variants and repetitive element insertions were not detected.

****Conclusions**:**

Tumor-only sequencing is adequate for the detection of clinically actionable germline variants, particularly for SNVs and small indels; however, a small subset of alterations affecting HRD, DDR and MMR genes may not be detected optimally. Therefore, for high-risk patients with negative tumor-only sequencing results, clinical genetic testing could be considered given the impact of these variants on therapy and genetic counseling.

## INTRODUCTION

The detection of pathogenic germline variants in cancer patients enables genetic counseling, risk-reducing measures and familial screening, as well as impacts the eligibility for genomically stratified clinical trials and the clinical use of targeted therapies.^1,2^ Deficiencies in DNA repair, including homologous recombination (HR), DNA damage response (DDR) and mismatch repair (MMR), have emerged as therapeutic targets,^3^ and the detection of actionable germline variants in genes pertaining to these biological processes is crucial to guide therapeutic decision making. Tumor-only sequencing has been proposed as an assay to detect germline variants alongside somatic variants.^4^ Whilst guidelines have been proposed to differentiate germline from somatic variants in tumor-only sequencing,^5^ this distinction in the absence of a matched normal sample remains challenging,^4^ based on technical and interpretational limitations inherent to this methodology.^6^ Here, we sought to assess the suitability of tumor-only sequencing for the detection of pathogenic/likely pathogenic (P/LP) germline variants affecting cancer susceptibility genes (CSGs), as compared to clinical germline testing in a cohort of >21 000 cancer patients, utilizing a Food and Drug Administration (FDA)-authorized and New York State Department of Health-approved tumor/normal sequencing assay and analytical pipeline.

## PATIENTS AND METHODS

### Study design

Our cohort included 21 333 cancer patients who had undergone tumor sequencing and genetic testing using the FDA-authorized Memorial Sloan Kettering-Integrated Mutation Profiling of Actionable Targets assay (MSK-IMPACT).^7^ New York State Department of Health-approved analytical pipelines were employed for the analysis of germline, tumor/normal pairs and tumor-only sequencing results. The detection of germline variants affecting moderate- and high-penetrance CSGs with autosomal dominant inheritance by tumor-only sequencing was compared to their detection in clinical germline testing using blood samples (See Supplementary Methods). To achieve a robust dataset, our analysis was restricted to CSGs with ≥15 P/LP unique germline variants in the cohort (Supplementary Table S1, available at https://doi.org/10.1016/j.annonc.2022.01.006). Sixteen genes were included in these analyses, including seven HR deficiency (HRD) genes (*BRCA2*, *BRCA1*, *PALB2*, *BRIP1*, *RAD51D*, *RAD51C* and *BAP1*), two DDR genes (*ATM* and *CHEK2*), four MMR genes (*MSH2*, *PMS2*, *MLH1* and *MSH6*) as well as *NF1*, *RB1* and *TP53*. Founder *BRCA1* (c.68_69delAG and c.5266dupC), *BRCA2* (c.5946delT), *CHEK2* (c.1100delC, c.1283C>T and c.470T>C), *MSH2* (c.1906G>C) and *MSH6* (c.3959_3962delCAAG and c.3984_3987dupGTCA) germline variants were excluded to avoid selection bias. Variant allele fraction (VAF) cut-offs put forward by the European Society of Medical Oncology (ESMO)^5^ were applied.

### Subjects and samples

This study was approved by the Memorial Sloan Kettering Cancer Center (MSKCC) Institutional Review Board (IRB), protocol 12–245 (Genomic profiling in cancer patients). Written informed patient consents were obtained as required by the approved IRB protocol. De-identified tumor and blood massively parallel sequencing data of 21 333 cancer patients enrolled on the institutional IRB-approved protocol 12–245 (NCT01775072) who underwent the FDA-authorized and New York State Department of Health-approved MSK-IMPACT sequencing between July 2015 and February 2021 were retrieved.

### Targeted capture-based sequencing

All individuals included in this study had a tumor and a paired blood sample sequenced using MSK-IMPACT,^7^ an FDA-authorized hybridization capture-based next-generation sequencing assay encompassing all protein-coding exons from the canonical transcripts and selected intronic and regulatory regions of >341 genes present across MSK-IMPACT versions. DNA was extracted from formalin-fixed paraffin-embedded tumor tissue and patient-matched blood samples, and DNA fragments were captured using custom probes, as previously described.^8^ Pooled libraries were sequenced on an Illumina (San Diego, CA) HiSeq 2500 with 2× 100 base-pair paired-end reads, as previously described.^8^

### Variant calling using matched tumor and blood MSK-IMPACT data

Sequencing reads were aligned to the human genome (hg19) using the Burrows-Wheeler Aligner (BWA; 0.7.5a) Reads were re-aligned around indels using ABRA (0.92),^9^ followed by base quality score recalibration with the Genome Analysis Toolkit (GATK) (3.3–0).^10^ Single-nucleotide variants (SNVs) were detected using MuTect^11^ and VarDict.^12^ Insertions and deletions were detected using SomaticIndelDetector^10^ and VarDict,^12^ as previously described.^8^ All mutations detected were re-genotyped in the tumor and patient-matched blood samples using only reads with mapping and base quality (MAPQ and BQ, respectively) ≥20. Mutations were annotated with VEP (v. 86).^13^ This analytical pipeline utilized has been authorized by the FDA and approved by the New York State Department of Health for clinical testing.

### Germline variant calling in blood samples

Germline variants were detected using MuTect, VarDict and GATK Haplotype caller as previously described.^14^ For certain genes with presence of highly homologous sequences, variant calling was carried out without a filter on MAPQ on sequencing reads. P/LP variants were determined and confirmed with an orthogonal assay before reporting to patients as per the New York State Department of Health guidelines. Copy number variants (CNVs) were identified using an in-house developed R script where GC content-corrected, coverage-normalized read counts for each target are used to identify gene-level and exon-level amplifications and deletions.^14^

### Variant calling using unmatched tumor-only MSK-IMPACT data

To simulate a tumor-only sequencing framework, we split the P/LP germline variants into two groups: (i) for single-nucleotide substitutions, we genotyped each variant in the tumor sample using reads with MAPQ ≥20; (ii) for large indels, we carried out unmatched variant calling on each tumor sample using a pooled control sample of DNA from 10 unrelated individuals as a comparator, as previously described.^8^ All results were analyzed by a molecular geneticist (PT). Results of called P/LP germline variants in each of the 16 CSGs obtained from tumor-only data were then compared to variants detected in the blood MSK-IMPACT data.

### Statistical analysis

The 95% confidence intervals (CIs) of percentages were computed using the Wilson procedure.

## RESULTS

**Figure 1.**
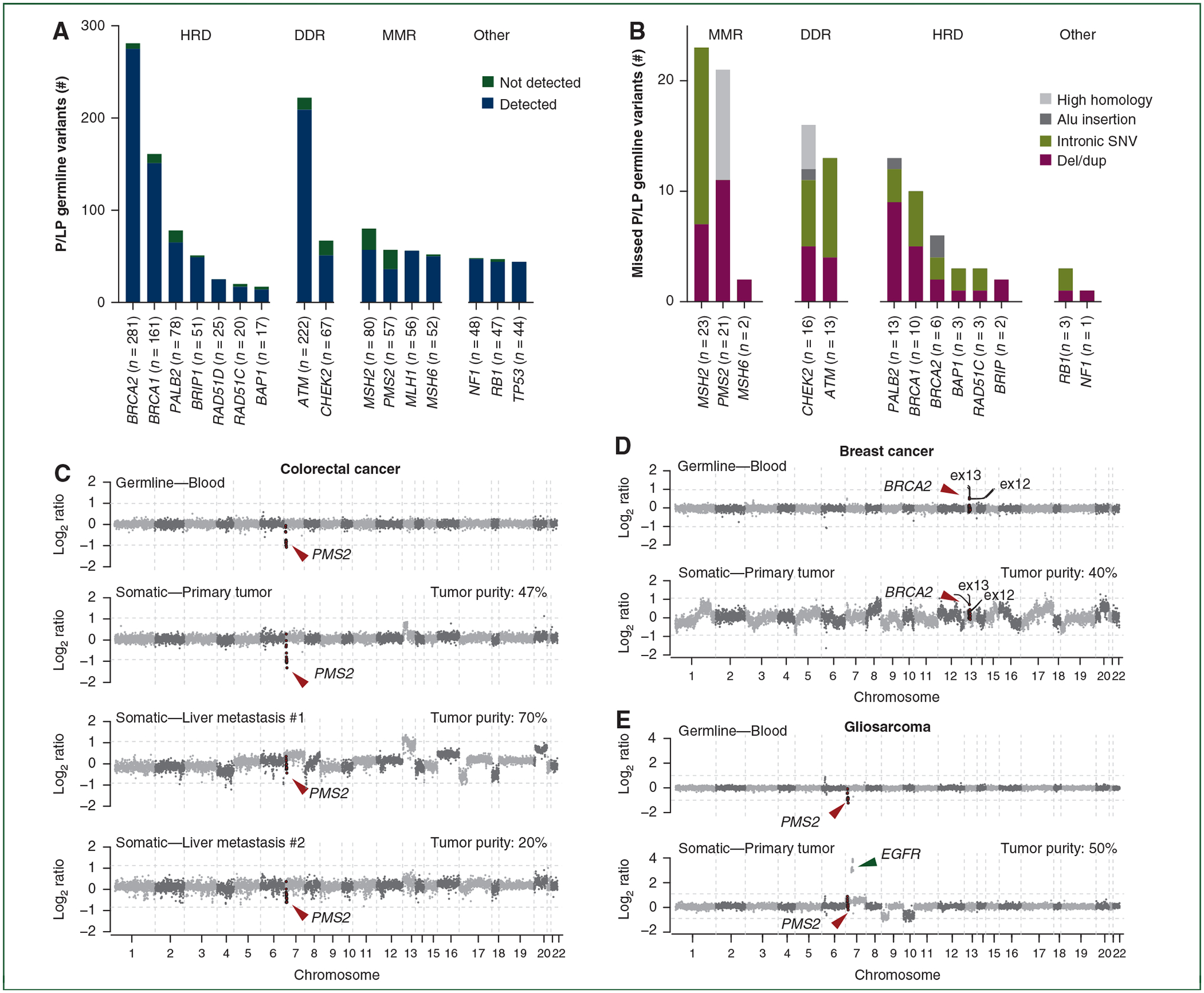
Technical detection of pathogenic/likely pathogenic (P/LP) germline variants affecting moderate- to high-penetrance cancer susceptibility genes by tumor-only sequencing. (A) P/LP germline variants technically not detected by tumor-only sequencing by gene and biological process. (B) P/LP germline variants not detected by tumor-only sequencing according to variant type. (C-E) Copy number plots depicting segmented Log_2_ ratios (*y*-axis) according to genomic position (*x*-axis) of blood and tumor samples in (C) a colorectal carcinoma with a *PMS2* germline whole gene deletion that is detectable in the primary tumor specimen but obscured due to genomic instability in two liver metastases, (D) a breast cancer with a germline *BRCA2* exon 12 and 13 duplication not detected in the tumor specimen due to somatic copy number variants (CNVs) and (E) a gliosarcoma with a germline *PMS2* whole gene deletion harboring a somatic *EGFR* amplification and chromosome 7 gain which prevents the detection of the germline *PMS2* deletion. HRD, homologous recombination deficiency; DDR, DNA damage response; MMR, mismatch repair; SNV, single-nucleotide variant.

Using the MSK-IMPACT platform, the median coverage for the genes included in this study was 582× (range 405 ×–737×) in the tumor specimen with 100% of exons with >100 ×coverage (Supplementary Figure S1, available at https://doi.org/10.1016/j.annonc.2022.01.006). We identified 1306 P/LP germline variants in blood or saliva samples affecting 16 moderate- and high-penetrance CSGs in 1282 cancer patients. 8.9% (95% CI 7.5% to 10.6%; 116/1306) of the P/LP germline variants were technically not detected by tumor-only sequencing. The P/LP germline variants technically not detected most frequently affected *PMS2* (36.8%; 21/57), *MSH2* (28.8%; 23/80), *CHEK2* (23.9%; 16/67), *BAP1* (17.6%; 3/17) and *PALB2* (16.7%; 13/78). Due to their relatively high prevalence, a substantial proportion of P/LP germline variants affecting *ATM* (13/222; 5.9%) and *BRCA1* (10/161; 6.2%) were also technically not detected (Figure 1A and Supplementary Table S2, available at https://doi.org/10.1016/j.annonc.2022.01.006). When the identical criteria were applied to 20 additional CSGs on the MSK-IMPACT panel with moderate penetrance and/or <15 pathogenic variants per gene, a similar proportion of technically not detected variants were identified with 8.2% (14/171) of variants not detected by tumor sequencing (Supplementary Figure S2, available at https://doi.org/10.1016/j.annonc.2022.01.006).

We next sought to determine whether the P/LP germline variants technically not detected by tumor-only sequencing were enriched for a particular variant type. Whilst all nonsense and missense SNVs and indels were detected by tumor-only sequencing, none of the intronic SNVs, Alu insertions or variants in regions of high homology, and only 54.9% of the deletion/duplications were identified (Figure 1B and Table 1). Notably, we observed that somatic copy number alterations and tumor sample quality frequently masked the germline variants investigated (Figure 1C–E).

**Figure 2.**
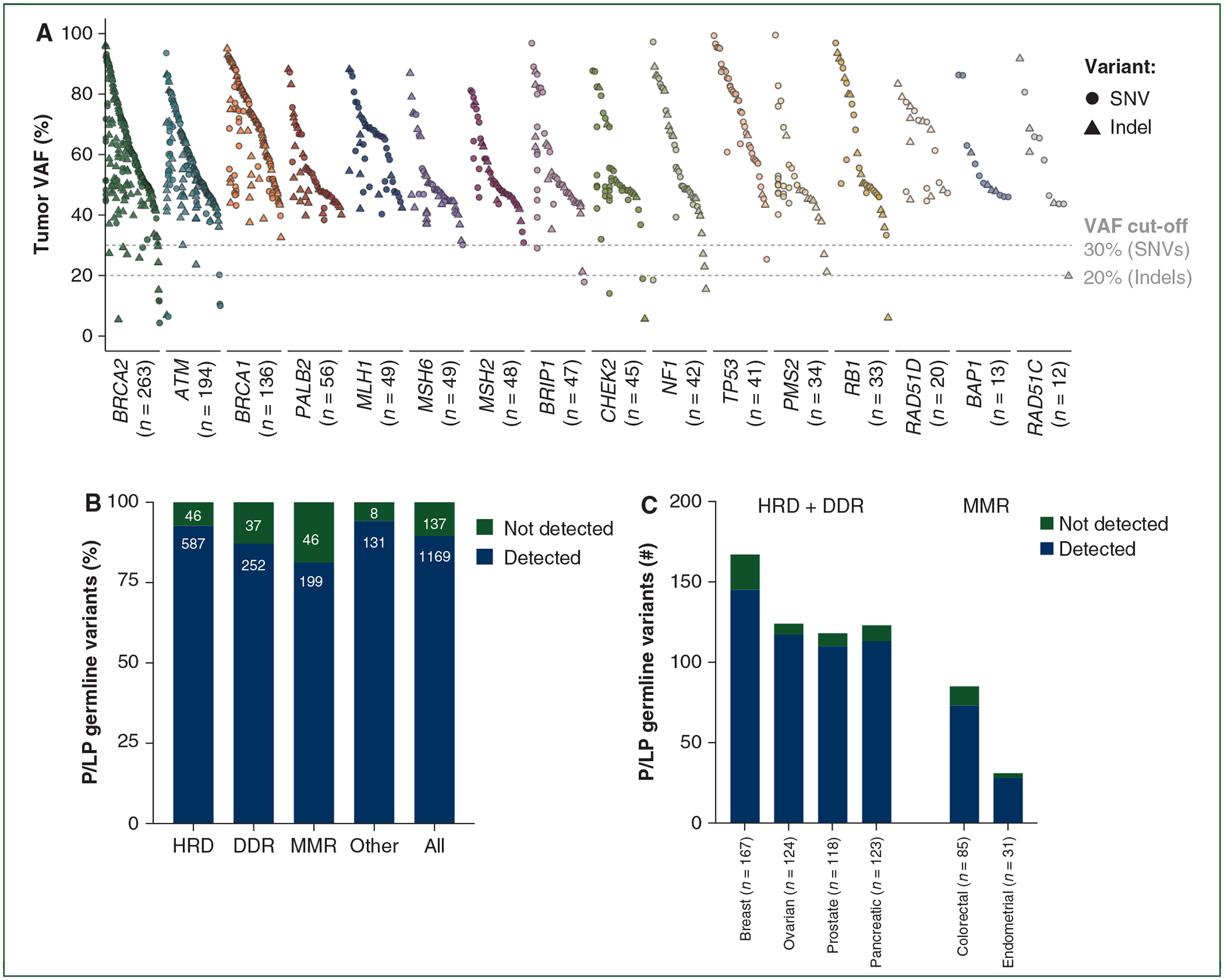
Variant allele fraction (VAF) of pathogenic/likely pathogenic (P/LP) germline variants and overall detection of germline variants as per biological process and tumor type. (A) Tumor VAF of the P/LP germline variants technically detected by tumor-only sequencing per cancer susceptibility gene (CSG). (B and C) Proportion of P/LP germline variants not detected by tumor-only sequencing either by technical detection or by a VAF lower than the recommended thresholds according to (B) biological process and (C) tumor type. HRD, homologous recombination deficiency; DDR, DNA damage response; MMR, mismatch repair; SNV, single-nucleotide variant.

A previous publication by the ESMO Precision Medicine Working Group recommended the adoption of VAF filters (i.e. VAF >20% for indels and VAF >30% for SNVs) to enrich for germline variants on tumor sequencing reports.^5^ Using these criteria, we observed that 21 (1.6%) additional variants were not detected by tumor-only sequencing due to a VAF in tumor lower than the recommended thresholds (Figure 2A and Supplementary Tables S3 and S4, available at https://doi.org/10.1016/j.annonc.2022.01.006). The ESMO study, however, only evaluated germline P/LP variants that would be reported on a tumor-only sequencing report. When the 8.9% of germline P/LP variants not included on tumor-only sequencing reports were added to the 1.6% of reported germline P/LP variants below VAF thresholds, we found in total that 10.5% (95% CI 8.9% to 12.3%; 137/1306) of P/LP germline variants in CSG were not detected by tumor-only sequencing resulting in an overall sensitivity of 89.5%. Of the P/LP germline variants targeting MMR, DDR and HRD genes, 18.8% (95% CI 14.4% to 24.1%; 46/245), 12.8% (95% CI 9.4% to 17.2%; 37/289) and 7.3% (95% CI 5.5% to 9.6%; 46/633), respectively, were not detected by tumor-only sequencing (Figure 2B and Table 2). These P/LP variants not detected could have significant clinical implications, in particular if the tumor-only sequencing assay employed cannot provide information about microsatellite instability high or HRD. Tumor-only sequencing was unable to detect 13.2% (95% CI 8.9% to 19.1%; 22/167), 8.1% (95% CI 4.5% to 14.3%; 10/123), 6.8% (95% CI 3.5% to 12.8%; 8/118) and 5.6% (95% CI 2.8% to 11.2%; 7/124) of the P/LP germline variants affecting HDR or DDR genes in breast, pancreatic, prostate and ovarian cancers, respectively, and 14.1% (95% CI 8.3% to 23.1%; 12/85) and 9.7% (95% CI 3.4% to 24.9%; 3/31) of the P/LP germline variants in MMR genes in colorectal and endometrial cancers, respectively (Supplementary Table S5, available at https://doi.org/10.1016/j.annonc.2022.01.006).

Some germline genetic testing guidelines including the UK National Institute for Health and Care Excellence recommend a pre-test probability of 10% to prompt genetic testing.^15^ Therefore, we applied two widely used prediction models, the PREMM5 model for Lynch syndrome risk^16^ and the Breast and Ovarian Analysis of Disease Incidence and Carrier Estimation Algorithm (BOADICEA) V5 model^17^ for HRD/DDR gene germline carrier risk (*ATM*, *CHEK2*, *BRCA1*, *BRCA2*, *BRIP1*, *PALB2*, *RAD51C* and *RAD51D*) to 126 patients with germline pathogenic variants in these genes. Using a 10% pre-test probability threshold, only 22% of HRD/DDR and 27% of Lynch syndrome germline pathogenic variant carriers whose mutations were not detected by tumor-only sequencing would have had germline genetic testing recommended by these models. If a more inclusive 5% pre-test probability threshold is used, 39% of HRD/DDR and 43% of Lynch syndrome pathogenic germline variant carriers whose mutations were not detected by tumor-only sequencing would have had germline genetic testing recommended by these models (Supplementary Figure S3, available at https://doi.org/10.1016/j.annonc.2022.01.006), demonstrating that only a subset of these germline pathogenic variants undetected by tumor-only sequencing would be captured by clinical genetics guidelines.

## DISCUSSION

Here, through the analysis of a large cohort of tumor/normal sequencing data, we demonstrate that a significant subset of actionable P/LP germline variants, including variants in HRD, DDR and MMR genes, are not detected by tumor-only sequencing. Poly (ADP-ribose) polymerase inhibitors are the current standard of care for various solid tumors harboring P/LP germline *BRCA1/2* variants, such as ovarian cancer^18^ and metastatic breast,^19^ prostate^20,21^ and pancreatic cancers,^22^ and may also benefit individuals with germline variants in HRD genes beyond *BRCA1/2*.^23,24^ Furthermore, DDR inhibitors, such as ATR and CHK1 inhibitors, have emerged as therapeutic agents for individuals with lesions in DDR genes, and clinical trials are ongoing.^25^ Hence, suboptimal detection of germline variants in HRD and DDR genes could significantly hinder therapeutic efforts in cancer patients. The high fraction of germline variants in MMR genes not detected by tumor-only sequencing that our analysis revealed could also be clinically detrimental to cancer patients’ families given that the family members would be unaware of their risk for Lynch syndrome and would not be screened under well-established Lynch syndrome protocols.^26^

Detection of germline variants based on tumor-only sequencing is fraught with difficulties. Variable tumor sample quality, somatic copy number alterations and intratumor genetic heterogeneity may mask exon-level CNVs (deletion/duplications) posing challenges for their detection. The design of tumor-only sequencing pipelines may also limit the ability to detect intronic variants other than those affecting consensus splice sites (i.e. ±2) as well as Alu insertions, variants in highly homologous regions and pseudogenes.^27,28^ Consistent with this notion, *PMS2*, *CHEK2* and *MSH2* P/LP germline variants were frequently not detected by tumor-only sequencing, given the enrichment of these genes for intronic SNVs and/or for deletion/duplications.

The 10.5% of germline pathogenic variants not detected by tumor-only sequencing in this study represent the likely true burden of germline variants undetected by tumor sequencing in clinical practice given that the FDA-authorized MSK-IMPACT assay has a high depth of coverage and a validated informatics pipeline that rivals those employed by commercial tumor sequencing assays. Moreover, the matched blood specimens for these patients were sequenced and analyzed to clinical genetic testing standards, far exceeding the quality metrics applied to publicly available tumor/normal sequencing databases such as The Cancer Genome Atlas (TCGA) or Pan-Cancer Analysis of Whole Genomes (PCAWG).

This study may suggest methodology changes that could improve the detection of germline pathogenic variants by tumor-only sequencing. For example, genotyping known pathogenic intronic variants through the addition of baits or primers capturing further into the intronic sequences where appropriate and/or modifying tumor sequencing informatics pipelines to be able to detect variants in regions of high homology would increase the detection rate. Likewise, more comprehensive tiling of the genomic loci of high-penetrance CSGs as well as more precise CNV detection algorithms may improve the detection rate for single- or multi-exon germline deletion or duplications. Our findings suggest that pipeline modifications of this nature could be considered if tumor-only sequencing is utilized as a screening approach for germline pathogenic variants.

Whilst tumor sequencing will not detect ~10% of germline pathogenic variants, it is worth noting that a robust workflow to flag potential germline pathogenic variant on tumor sequencing reports can substantially decrease the probability of carrying a germline pathogenic variant and potentially be a cost-effective methodology. For example, for breast cancer, 7.1% (167/2353) of patients carried a germline pathogenic variant in a DDR or HRD gene. If all germline pathogenic variants appearing on tumor-only sequencing reports were identified as germline in origin, 0.9% (22/2353) of breast cancer patients would remain with undetected germline pathogenic variants (Supplementary Table S5, available at https://doi.org/10. 1016/j.annonc.2022.01.006). Although these numbers are encouraging, even a 0.9% false-negative rate would affect a substantial number of individuals, given that >2 million new cases of breast cancer are diagnosed each year worldwide. Extrapolating our findings to the number of individuals diagnosed with breast cancer on a yearly basis, 19 541 (95% CI 12 749–29 469) breast cancer patients worldwide could potentially carry a P/LP germline variant affecting HRD or DDR genes that would be not be detected by tumor-only sequencing (Supplementary Table S5, available at https://doi.org/10.1016/j.annonc.2022.01.006). Coupling genetic pre-test probability calculations with tumor-only sequencing, however, does provide an additive benefit and can be considered for high-risk patients with tumor sequencing reports negative for the gene of interest. For example, applying the BOADICEA V5 model for HRD/DDR gene germline carrier risk (*ATM*, *CHEK2*, *BRCA1*, *BRCA2*, *BRIP1*, *PALB2*, *RAD51C* and *RAD51D*) to the 22 breast cancer patients with germline pathogenic variants in these genes not detected by tumor-only sequencing, only 10 patients fell below the 10% pre-test probability cut-off, whereas 4 fell below the 5% cut-off. This would translate to 0.4% and 0.2% of breast cancer patients with undetected germline pathogenic variants by tumor-only sequencing. Hence, genetic pre-test probability calculations can help capture a subset of the P/LP variants not detected by tumor-only sequencing through reflex germline testing, and can incrementally add to the detection rate of germline pathogenic variants following a tumor-only sequencing report that would be negative for these variants. Our findings, however, are consistent with the notion that clinical germline testing for cancer patients still remains the ‘gold standard’ for the detection of these variants.

### Limitations

Our study has important limitations. Our analysis was restricted to MSK-IMPACT genes and our cohort included a limited number of germline variants for each CSG studied. We were unable to ascertain whether the observations made in this study would be applicable to other sequencing assays, given that our study was predicated on the quantification of the burden of germline variants technically not detected in tumor-only sequencing using a clinically validated assay to define the clinical implications in the context of a ‘real-world’ tumor sequencing report. The somatic and germline laboratory methodologies and bioinformatics pipelines used in our analysis have been through extensive clinical validation and New York State Department of Health approval, and the sequencing was carried out with an FDA-authorized sequencing assay. This type of technical rigor cannot be replicated in publicly available datasets (e.g. those from TCGA or PCAWG datasets), given that (i) the sequencing was carried out in a research setting with research approaches for library preparations and massively parallel sequencing; (ii) the depth of sequencing carried out by TCGA or PCAWG would not meet the standards required for a clinical assay; (iii) the analytical pipelines utilized in those studies are ‘research only’ and would not necessarily represent the practice in a ‘real-world’ setting in terms of stringency and types of variants detected; and (iv) the subset of cases from the Genomics Evidence Neoplasia Information Exchange (GENIE) dataset with tumor/normal sequencing, which could be employed to validate the findings of this study, largely stem from MSKCC and were derived using MSK-IMPACT. Based on the above, our findings represent a conservative estimate (i.e. best-case scenario) of the variants technically not detected by tumor-only sequencing, given that the same sequencing assay (i.e. MSK-IMPACT) was employed for both tumor-only sequencing and for the germline sequencing.

### Conclusions

Most actionable germline variants, including variants affecting HRD, DDR and MMR genes, are detected and reported by tumor-only sequencing. While tumor-only sequencing is adequate for the detection of pathogenic germline variants, a small subset of actionable germline variants is currently not detected by this approach. Hence, pipeline and workflow modifications that would substantially improve the detection rate might be considered. Depending on health economics, local resources and infrastructure, systematic tumor-normal sequencing or clinical germline sequencing of cancer patients might be entertained, at least for high-risk patients.

## Supplementary Material

supplementary material

## Figures and Tables

**Table 1.** Pathogenic/likely pathogenic germline variants not detected by tumor-only sequencing by lack of technical detection according to variant type

CSG	Nonsense or splice site SNV		Missense SNV		Indel <5 bp		Indel >5 bp		Deletion/duplication		Intronic SNV		Alu insertion		High homology		Total	
	Total	Not detected, *n* (%)	Total	Not detected, *n* (%)	Total	Not detected, *n* (%)	Total	Not detected, *n* (%)	Total	Not detected, *n* (%)	Total	Not detected, *n* (%)	Total	Not detected, *n* (%)	Total	Not detected, *n* (%)	Total	Not detected, *n* (%)
*BRCA1*	45	0 (0.0)	30	0 (0.0)	58	0 (0.0)	10	0 (0.0)	13	5 (38.5)	5	5 (100.0)	0	NA	0	NA	161	10 (6.2)
*BRCA2*	74	0 (0.0)	16	0 (0.0)	158	0 (0.0)	26	0 (0.0)	3	2 (66.7)	2	2 (100.0)	2	2 (100.0)	0	NA	281	6 (2.1)
*NF1*	16	0 (0.0)	5	0 (0.0)	18	0 (0.0)	3	0 (0.0)	6	1 (16.7)	0	NA	0	NA	0	NA	48	1 (2.1)
*PALB2*	21	0 (0.0)	2	0 (0.0)	32	0 (0.0)	5	0 (0.0)	14	9 (64.3)	3	3 (100.0)	1	1 (100.0)	0	NA	78	13 (16.7)
*ATM*	83	0 (0.0)	21	0 (0.0)	82	0 (0.0)	15	0 (0.0)	12	4 (33.3)	9	9 (100.0)	0	NA	0	NA	222	13 (5.9)
*BRIP1*	26	0 (0.0)	2	0 (0.0)	19	0 (0.0)	1	0 (0.0)	3	2 (66.7)	0	NA	0	NA	0	NA	51	2 (3.9)
*CHEK2*	24	0 (0.0)	11	0 (0.0)	9	0 (0.0)	1	0 (0.0)	11	5 (45.5)	6	6 (100.0)	1	1 (100.0)	4	4 (100.0)	67	16 (23.9)
*MLH1*	22	0 (0.0)	13	0 (0.0)	16	0 (0.0)	0	NA	5	0 (0.0)	0	NA	0	NA	0	NA	56	0 (0.0)
*MSH2*	23	0 (0.0)	12	0 (0.0)	10	0 (0.0)	3	0 (0.0)	16	7 (43.8)	16	16 (100.0)	0	NA	0	NA	80	23 (28.8)
*MSH6*	10	0 (0.0)	5	0 (0.0)	32	0 (0.0)	3	0 (0.0)	2	2 (100.0)	0	NA	0	NA	0	0	52	2 (3.8)
*PMS2*	14	0 (0.0)	11	0 (0.0)	9	0 (0.0)	1	0 (0.0)	12	11 (91.7)	0	NA	0	NA	10	10 (100.0)	57	21 (36.8)
*RB1*	19	0 (0.0)	3	0 (0.0)	13	0 (0.0)	3	0 (0.0)	7	1 (14.3)	2	2 (100.0)	0	NA	0	NA	47	3 (6.4)
*TP53*	9	0 (0.0)	29	0 (0.0)	3	0 (0.0)	1	0 (0.0)	2	0 (0.0)	0	NA	0	NA	0	NA	44	0 (0.0)
*BAP1*	7	0 (0.0)	2	0 (0.0)	3	0 (0.0)	1	0 (0.0)	2	1 (50.0)	2	2 (100.0)	0	NA	0	NA	17	3 (17.6)
*RAD51C*	8	0 (0.0)	2	0 (0.0)	5	0 (0.0)	1	0 (0.0)	2	1 (50.0)	2	2 (100.0)	0	NA	0	NA	20	3 (15.0)
*RAD51D*	11	0 (0.0)	1	0 (0.0)	9	0 (0.0)	1	0 (0.0)	3	0 (0.0)	0	NA	0	NA	0	NA	25	0 (0.0)
Total	412	0 (0.0)	165	0 (0.0)	476	0 (0.0)	75	0 (0.0)	113	51 (45.1)	47	47 (100.0)	4	4 (100)	14	14 (100)	1306	116 (8.9)

CSG, cancer susceptibility gene; NA, not applicable; SNV, single-nucleotide variant.

**Table 2.** Pathogenic/likely pathogenic germline variants nor detected by tumor-only sequencing according to biological process

Biological process	Total	Detected	Not detected			
			*n*	%	Lower 95% CI	Upper 95% CI
HRD	633	587	46	7.3	5.5%	9.6%
DDR	289	252	37	12.8	9.4%	17.2%
MMR	245	199	46	18.8	14.4%	24.1%
Other	139	131	8	5.8	3.0%	11.0%
Total	1306	1169	137	10.5	8.9%	12.3%

CI, confidence interval; HRD, homologous recombination deficiency; DDR, DNA damage response; MMR, mismatch repair.
