# Supplementary material for: Comprehensive assessment of germline pathogenic variant detection in tumor-only sequencing
Source: Ann Oncol. Author manuscript; Available in PMC 2022 Jun 7. (PMC9172914; doi:10.1016/j.annonc.2022.01.006)

**SUPPLEMENTARY MATERIALS**

**Comprehensive assessment of germline pathogenic variant detection in tumor-only sequencing**

Terraf et al.

**Supplementary Methods**

**Supplementary Table S1.** Moderate and high penetrance cancer susceptibility genes from the MSK-IMPACT panel included in this study

**Supplementary Figure S1:** Median sequencing coverage for 21,333 tumor specimens in this cohort

**Supplementary Figure S2**: Technical detection of pathogenic/likely pathogenic (P/LP) germline variants affecting moderate penetrance CSGs or CSGs with <15 pathogenic germline variants by tumor-only sequencing.

**Supplementary Table S2.** Pathogenic/ likely pathogenic germline variants not detected by tumor-only sequencing

**Supplementary Table S3.** Pathogenic/ likely pathogenic germline variants not detected by tumor-only sequencing due to a VAF lower than the recommended thresholds

**Supplementary Table S4**. Pathogenic/ likely pathogenic germline variants in additional CSG’s not detected by tumor-only sequencing due to a VAF lower than the recommended thresholds

**Supplementary Table S5.** Pathogenic/likely pathogenic germline variants not detected by tumor-only testing by technical lack of detection or by a VAF below the recommended thresholds according to tumor type and biological process

**Supplementary Figure S3:** Pre-test probability of pathogenic germline variant carrier status for 108 cancer patients with variants not detected by tumor-only sequencing as calculated using the BOADICEA V5 (HDR/DDR) or PREMM5 (Lynch Syndrome) models

**SUPPLEMENTARY METHODS**

**Subjects and samples**

This study was approved by Memorial Sloan Kettering Cancer Center (MSKCC; NY, USA) Institutional Review Board (IRB), protocol 12-245 (Genomic profiling in cancer patients). Written informed patient consents were obtained as required by the approved IRB protocol. De-identified tumor and blood massively parallel sequencing data of 21,333 cancer patients enrolled on the institutional IRB approved protocol 12-245 (NCT01775072) who underwent the Fda-approved MSK-Integrated Mutation Profiling of Actionable Targets (MSK-IMPACT) sequencing between July 2015 and February 2021 were retrieved.

**Targeted capture-based sequencing**

All individuals included in this study had a tumor and a paired blood sample sequenced using MSK-IMPACT [1], a Food and Drug Administration-authorized hybridization capture-based next-generation sequencing assay encompassing all protein-coding exons from the canonical transcript of >341 genes present across MSK-IMPACT versions. DNA was extracted from formalin-fixed paraffin-embedded tumor tissue and patient-matched blood samples, and DNA fragments were captured using custom probes, as previously described [2]. Pooled libraries were sequenced on an Illumina HiSeq 2500 with 2 × 100-base-pair paired-end reads, as previously described [2].

**Variant calling using matched tumor and blood MSK-IMPACT data**

Sequencing reads were aligned to the human genome (hg19) using BWA (0.7.5a). Reads were re-aligned around indels using ABRA (0.92) [3], followed by base quality score recalibration with the Genome Analysis Toolkit (GATK) (3.3-0) [4]. Single nucleotide variants (SNVs) were detected using Mutect [5] and VarDict [6]. Insertions and deletions were detected using SomaticIndelDetector [4] and VarDict [6], as previously described [2]. All called mutations were re-genotyped in the tumor and patient-matched blood sample using only reads with mapping and base quality (MAPQ and BQ, respectively) ≥ 20. Mutations were annotated with VEP (v.86) [7].

**Germline variant calling in blood samples**

Germline variants were detected using VarDict and GATK Haplotype caller as previously described [8]. For certain genes with presence of highly homologous sequences, variant calling was performed without a filter on MAPQ on sequencing reads. P/LP variants were determined and confirmed with an orthogonal assay before reporting to patients per New York State Department of Health guidelines. Copy number alterations were identified using an in-house developed R script where GC-content corrected, coverage normalized read counts for each target are used to identify gene level and exon level amplifications and deletions [8].

**Variant calling using unmatched tumor-only MSK-IMPACT data**

To simulate a tumor-only sequencing framework, we split the P/LP germline variants into two groups : i) for single nucleotide substitutions we genotyped each variant in the tumor sample using reads with MAPQ ≥ 20 ii) for large indels, we performed unmatched variant calling on each tumor sample using a pooled control sample of DNA from ten unrelated individuals as a comparator, as previously described[2]. All results were analyzed by a molecular geneticist (PT). Results of called P/LP germline variants in each of the 16 cancer susceptibility genes obtained from tumor-only data were then compared to called variants from the blood MSK-IMPACT data. Founder *BRCA1* (c.68_69delAG and c.5266dupC), *BRCA2* (c.5946delT), *CHEK2* (c.1100delC, c.1283C>T and c.470T>C), *MSH2* (c.1906G>C) and *MSH6* (c.3959_3962delCAAG and c.3984_3987dupGTCA) variants were excluded from the analysis to avoid selection bias.

**Statistical analysis**

The 95% confidence intervals of percentages were computed using the Wilson procedure.

**Determination of genetic variant carrier pre-test probability**

In order to assess the pre-test probability of germline pathogenic variants in HRD/DDR (ATM, CHEK2, BRCA1, BRCA2, BRIP1, PALB2, RAD51C and RAD51D) and MMR (MLH1, MSH2, MSH6, PMS2, or EPCAM) genes in individuals who had negative tumor-only testing, two clinical prediction models (i.e. BOADICEA and PREMM5, respectively) were applied to 126 patients with germline pathogenic variants in these genes. Both models use information on the individual being evaluated such as prior cancer diagnoses, lifestyle and hormonal risk factors as well as family history, including types of cancer and ages at diagnosis of first- and second-degree relatives to estimate the cumulative probability of a germline mutation in the aforementioned genes.

**SUPPLEMENTARY REFERENCES**

1. Cheng DT, Mitchell TN, Zehir A et al. Memorial Sloan Kettering-Integrated Mutation Profiling of Actionable Cancer Targets (MSK-IMPACT): A Hybridization Capture-Based Next-Generation Sequencing Clinical Assay for Solid Tumor Molecular Oncology. J Mol Diagn 2015; 17: 251-264.

2. Bolton KL, Ptashkin RN, Gao T et al. Cancer therapy shapes the fitness landscape of clonal hematopoiesis. Nat Genet 2020; 52: 1219-1226.

3. Mose LE, Wilkerson MD, Hayes DN et al. ABRA: improved coding indel detection via assembly-based realignment. Bioinformatics 2014; 30: 2813-2815.

4. McKenna A, Hanna M, Banks E et al. The Genome Analysis Toolkit: a MapReduce framework for analyzing next-generation DNA sequencing data. Genome Res 2010; 20: 1297-1303.

5. Cibulskis K, Lawrence MS, Carter SL et al. Sensitive detection of somatic point mutations in impure and heterogeneous cancer samples. Nat Biotechnol 2013; 31: 213-219.

6. Lai Z, Markovets A, Ahdesmaki M et al. VarDict: a novel and versatile variant caller for next-generation sequencing in cancer research. Nucleic Acids Res 2016; 44: e108.

7. McLaren W, Gil L, Hunt SE et al. The Ensembl Variant Effect Predictor. Genome Biol 2016; 17: 122.

8. Cheng DT, Prasad M, Chekaluk Y et al. Comprehensive detection of germline variants by MSK-IMPACT, a clinical diagnostic platform for solid tumor molecular oncology and concurrent cancer predisposition testing. BMC Med Genomics 2017; 10: 33.

**Supplementary Table S1. Moderate and high penetrance cancer susceptibility genes from the MSK-IMPACT panel included in this study**

| **Genes** | **Diseases/Syndromes** | **Included in analysis** | **Number of P/LP germline variants** |
| --- | --- | --- | --- |
| *APC* | Familial adenomatous polyposis | No | NA |
| *ATM* | Ataxia-telangiectasia; ATM-related cancer risk | Yes | 222 |
| *BAP1* | Mesothelioma, uveal melanoma, renal cell carcinoma | Yes | 17 |
| *BARD1* | Hereditary breast and ovarian cancer syndrome | No | NA |
| *BRCA1* | Hereditary breast and ovarian cancer syndrome | Yes | 161 |
| *BRCA2* | Hereditary breast and ovarian cancer syndrome; Fanconi anemia | Yes | 281 |
| *BRIP1* | BRIP1-related cancer; Fanconi anemia | Yes | 51 |
| *CDH1* | Hereditary diffuse gastric cancer | No | NA |
| *CDK4* | Familial cutaneous melanoma | No | NA |
| *CDKN2A* | Familial cutaneous melanoma | No | NA |
| *CHEK2* | CHEK2-related cancer | Yes | 67 |
| *DICER1* | DICER1-Related Disorders. | No | NA |
| *FH* | Hereditary Leiomyomatosis and Renal Cell Cancer | No | NA |
| *FLCN* | Birt-Hogg-Dubé syndrome | No | NA |
| *MEN1* | Multiple endocrine neoplasia, type 1 | No | NA |
| *MLH1* | Lynch syndrome | Yes | 56 |
| *MSH2* | Lynch syndrome | Yes | 100 |
| *MSH6* | Lynch syndrome | Yes | 67 |
| *NBN* | Nijmegen breakage syndrome; NBN-related cancer risk | No | NA |
| *NF1* | Neurofibromatosis, type 1 | Yes | 48 |
| *NF2* | Neurofibromatosis, type 2 | No | NA |
| *PALB2* | PALB2-related cancer; Fanconi anemia | Yes | 78 |
| *PMS2* | Lynch syndrome | Yes | 57 |
| *RAD51C* | RAD51C-related cancer; Fanconi anemia | Yes | 20 |
| *RAD51D* | Hereditary ovarian cancer | Yes | 25 |
| *RB1* | Retinoblastoma | Yes | 47 |
| *RET* | Multiple endocrine neoplasia, type 2 | No | NA |
| *SDHA* | Hereditary paraganglioma-pheochromocytoma (PGL/PCC) syndromes | No | NA |
| *SDHAF2* | Hereditary paraganglioma-pheochromocytoma (PGL/PCC) syndromes | No | NA |
| *SDHB* | Hereditary paraganglioma-pheochromocytoma (PGL/PCC) syndromes | No | NA |
| *SDHC* | Hereditary paraganglioma-pheochromocytoma (PGL/PCC) syndromes | No | NA |
| *SDHD* | Hereditary paraganglioma-pheochromocytoma (PGL/PCC) syndromes | No | NA |
| *SMAD4* | Juvenile polyposis syndrome | No | NA |
| *SMARCA4* | Rhabdoid tumor predisposition syndrome type 2 | No | NA |
| *SMARCB1* | Rhabdoid tumor predisposition syndrome type 1 | No | NA |
| *STK11* | Peutz-Jeghers syndrome | No | NA |
| *SUFU* | Medulloblastoma | No | NA |
| *TERT* | Familial pulmonary fibrosis (FPF); Dyskeratosis congenita (DC) | No | NA |
| *TP53* | Li-Fraumeni syndrome | Yes | 44 |
| *TSC1* | Tuberous sclerosis complex (TSC) | No | NA |
| *TSC2* | Tuberous sclerosis complex (TSC) | No | NA |
| *VHL* | Von Hippel-Lindau syndrome; Familial erythrocytosis, type 2 | No | NA |

NA, not applicable

**Supplementary Figure S1: Median sequencing coverage for 21,333 tumor specimens in this cohort**


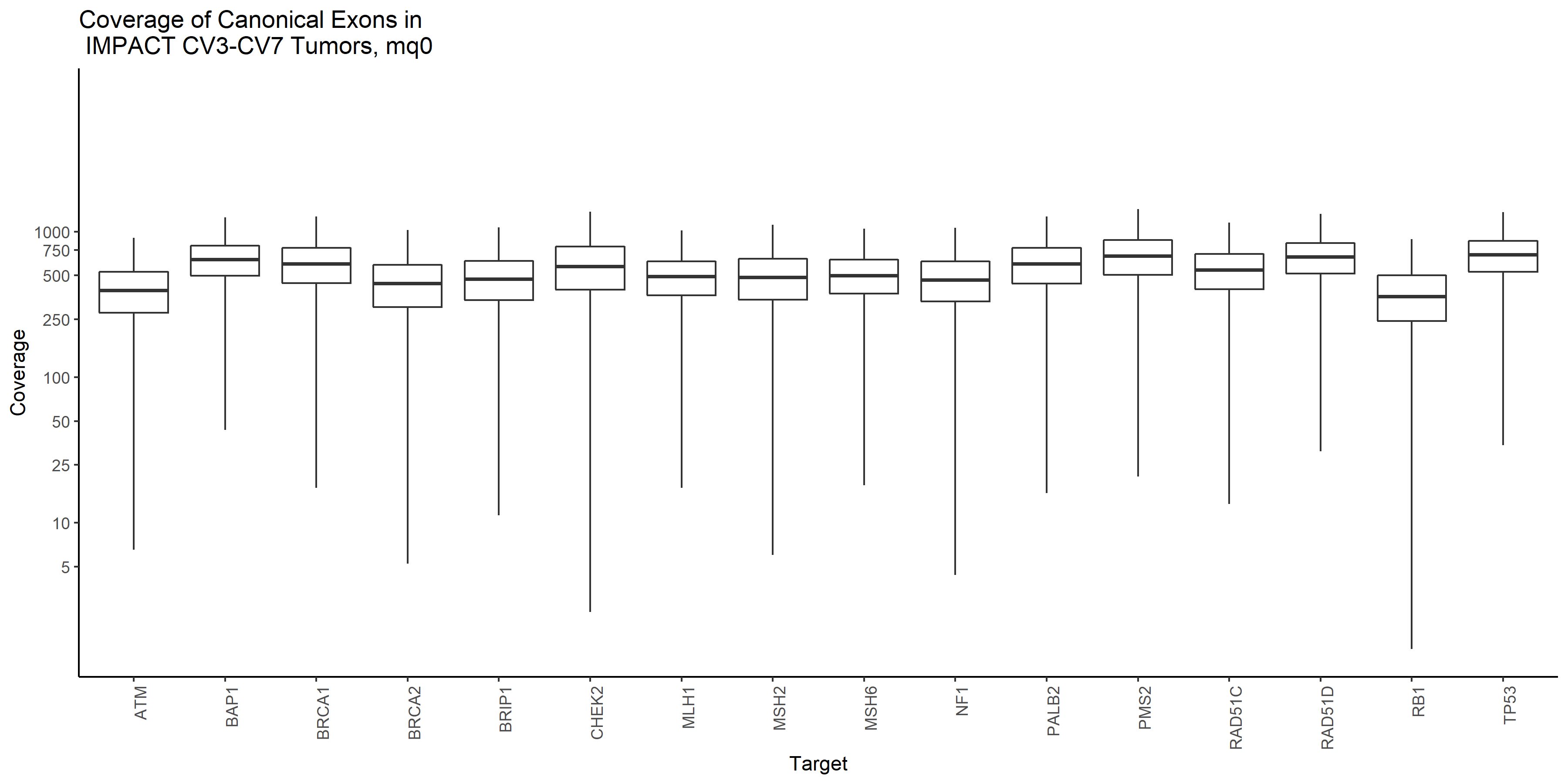


The median sequencing coverage and range is presented for the 16 genes in the analysis

**Supplementary Figure S2: Technical detection of pathogenic/likely pathogenic (P/LP) germline variants affecting moderate penetrance CSGs or CSGs with <15 pathogenic germline variants by tumor-only sequencing.**


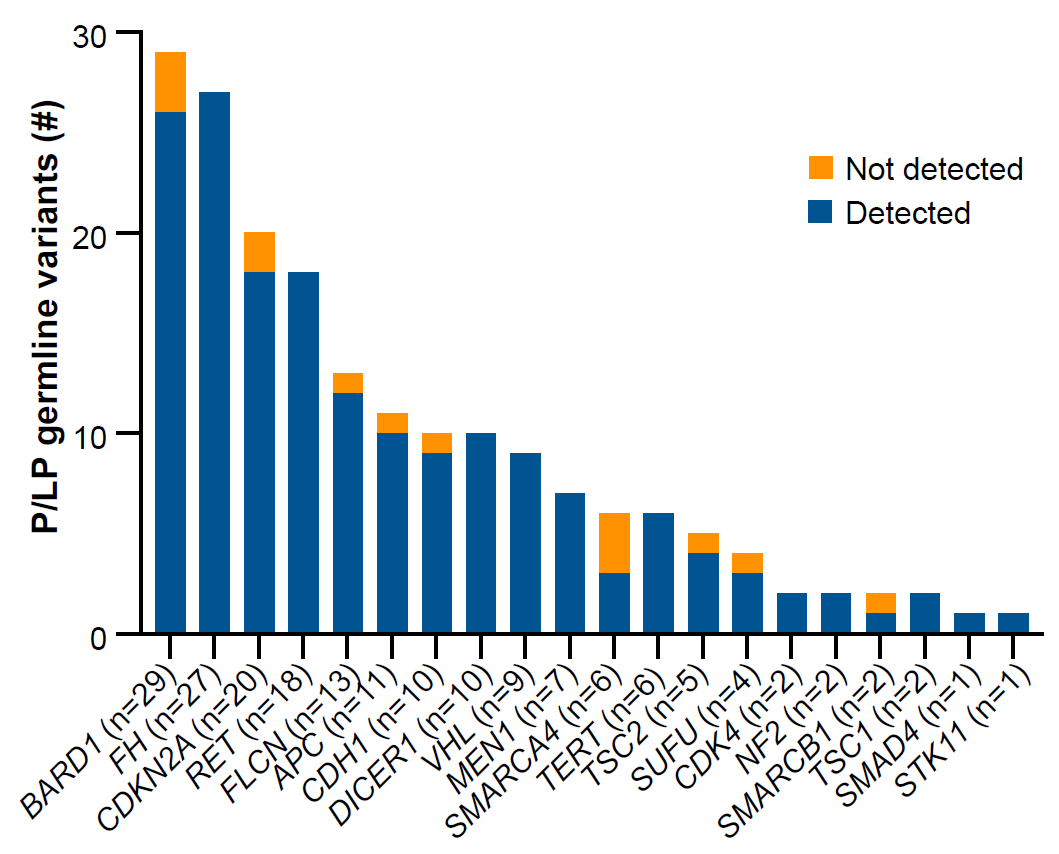


P/LP germline variants technically not detected by tumor-only sequencing by gene.

**Supplementary Table S2.** **Pathogenic/ likely pathogenic germline variants not detected by tumor-only sequencing.**

| **CSG** | **Biological process** | **Total germline P/LP variants** | **Variants not detected technically** | | | | **Variants not detected by low VAF** | | | | **Variants not detected technically or by VAF threshold** | | | |
| --- | --- | --- | --- | --- | --- | --- | --- | --- | --- | --- | --- | --- | --- | --- |
|  |  |  | **Number** | **%** | **Lower 95 CI** | **Upper 95 CI** | **Number** | **%** | **Lower 95 CI** | **Upper 95 CI** | **Number** | **%** | **Lower 95 CI** | **Upper 95 CI** |
| *BRCA2* | HRD | 281 | 6 | 2.1% | 1.0% | 4.6% | 6 | 2.1% | 1.0% | 4.6% | 12 | 4.3% | 2.5% | 7.3% |
| *BRCA1* | HRD | 161 | 10 | 6.2% | 3.4% | 11.1% | 0 | 0.0% | 0.0% | 0.0% | 10 | 6.2% | 3.4% | 11.1% |
| *NF1* | Other | 48 | 1 | 2.1% | 0.4% | 10.9% | 2 | 4.2% | 1.2% | 14.0% | 3 | 6.3% | 2.2% | 16.8% |
| *PALB2* | HRD | 78 | 13 | 16.7% | 10.0% | 26.5% | 0 | 0.0% | 0.0% | 0.0% | 13 | #### | 10.0% | 26.5% |
| *ATM* | DDR | 222 | 13 | 5.9% | 3.5% | 9.8% | 5 | 2.3% | 1.0% | 5.2% | 18 | 8.1% | 5.2% | 12.5% |
| *BRIP1* | HRD | 51 | 2 | 3.9% | 1.1% | 13.2% | 2 | 3.9% | 1.1% | 13.2% | 4 | 7.8% | 3.1% | 18.5% |
| *CHEK2* | DDR | 67 | 16 | 23.9% | 15.3% | 35.3% | 3 | 4.5% | 1.5% | 12.4% | 19 | #### | 19.0% | 40.1% |
| *MSH2* | MMR | 80 | 23 | 28.8% | 20.0% | 39.5% | 0 | 0.0% | 0.0% | 0.0% | 23 | #### | 20.0% | 39.5% |
| *PMS2* | MMR | 57 | 21 | 36.8% | 25.5% | 49.8% | 0 | 0.0% | 0.0% | 0.0% | 21 | #### | 25.5% | 49.8% |
| *MLH1* | MMR | 56 | 0 | 0.0% | 0.0% | 0.0% | 0 | 0.0% | 0.0% | 0.0% | 0 | 0.0% | 0.0% | 0.0% |
| *MSH6* | MMR | 52 | 2 | 3.8% | 1.1% | 13.0% | 0 | 0.0% | 0.0% | 0.0% | 2 | 3.8% | 1.1% | 13.0% |
| *RB1* | Other | 47 | 3 | 6.4% | 2.2% | 17.2% | 1 | 2.1% | 0.4% | 11.1% | 4 | 8.5% | 3.4% | 19.9% |
| *TP53* | Other | 44 | 0 | 0.0% | 0.0% | 0.0% | 1 | 2.3% | 0.4% | 11.8% | 1 | 2.3% | 0.4% | 11.8% |
| *RAD51D* | HRD | 25 | 0 | 0.0% | 0.0% | 0.0% | 0 | 0.0% | 0.0% | 0.0% | 0 | 0.0% | 0.0% | 0.0% |
| *RAD51C* | HRD | 20 | 3 | 15.0% | 5.2% | 36.0% | 1 | 5.0% | 0.9% | 23.6% | 4 | #### | 8.1% | 41.6% |
| *BAP1* | HRD | 17 | 3 | 17.6% | 6.2% | 41.0% | 0 | 0.0% | 0.0% | 0.0% | 3 | #### | 6.2% | 41.0% |
| Total | - | 1306 | 116 | 8.9% | 7.5% | 10.6% | 21 | 1.6% | 1.1% | 2.5% | 137 | #### | 8.9% | 12.3% |

**Supplementary Table S3. Pathogenic/ likely pathogenic germline variants not detected by tumor-only sequencing due to a VAF lower than the recommended thresholds.**

| **CSG** | **Germline variant (protein change)** | **Variant type** | **VAF (Blood)** | **VAF (Tumor)** |
| --- | --- | --- | --- | --- |
| *BRCA2* | c.6037A>T(p.Lys2013*) | SNV | 0.47512 | 0.29245 |
| *BRIP1* | c.2392C>T(p.Arg798*) | SNV | 0.46786 | 0.29043 |
| *TP53* | c.542G>A(p.Arg181His) | SNV | 0.49307 | 0.25327 |
| *ATM* | c.748C>T(p.Arg250*) | SNV | 0.46172 | 0.20204 |
| *RAD51C* | c.394dupA(p.Thr132Asnfs*23) | Indel | 0.46254 | 0.19811 |
| *CHEK2* | c.444+1G>A() | SNV | 0.49421 | 0.18922 |
| *NF1* | c.1885G>A(p.Gly629Arg) | SNV | 0.314 | 0.185 |
| *BRIP1* | c.103G>T(p.Gly35*) | SNV | 0.51834 | 0.17848 |
| *NF1* | c.2158_2177dupCGGTGTGGGGTGGATGAAGT(p.Ser727Glyfs*28) | Indel | 0.11088 | 0.15471 |
| *BRCA2* | c.8673_8674delAA(p.Arg2892Thrfs*14) | Indel | 0.47887 | 0.15194 |
| *CHEK2* | c.444+1G>A() | SNV | 0.43762 | 0.14058 |
| *BRCA2* | c.9466C>T(p.Gln3156*) | SNV | 0.35028 | 0.11686 |
| *BRCA2* | c.7718T>G(p.Leu2573*) | SNV | 0.53298 | 0.11538 |
| *ATM* | c.8473C>T(p.Gln2825*) | SNV | 0.49738 | 0.10526 |
| *ATM* | c.7775C>G(p.Ser2592Cys) | SNV | 0.28685 | 0.1 |
| *ATM* | c.1027_1030delGAAA(p.Glu343Ilefs*2) | Indel | 0.435 | 0.068 |
| *ATM* | c.7913G>A(p.Trp2638*) | SNV | 0.49689 | 0.06422 |
| *RB1* | c.2265_2343delinsAT(p.?) | Indel | 0.332 | 0.06 |
| *CHEK2* | c.276delC(p.Trp93Glyfs*17) | Indel | 0.32267 | 0.05609 |
| *BRCA2* | c.5073dupA(p.Trp1692Metfs*3) | Indel | 0.41606 | 0.05426 |
| *BRCA2* | c.7558C>T(p.Arg2520*) | SNV | 0.48458 | 0.0431 |

**Supplementary Table S4. Pathogenic/ likely pathogenic germline variants in additional CSGs not detected by tumor-only sequencing due to a VAF lower than the recommended thresholds.**

| **CSG** | **Germline variant (protein change)** | **Variant type** | **VAF (Blood)** | **VAF (Tumor)** |
| --- | --- | --- | --- | --- |
| *APC* | c.3798_3817dupTACTCCAATATGTTTTTCAA (p.Arg1273Ilefs*22) | Indel | 0.23885 | 0.16892 |
| *BARD1* | c.627_628delAA (p.Lys209Asnfs*4) | Indel | 0.43516 | 0.13720 |
| *CDH1* | c.376_382dupCCGCCCC (p.His128Profs*42) | Indel | 0.18129 | 0.12586 |
| *DICER1* | c.2045delC (p.Pro682Hisfs*3) | Indel | 0.39879 | 0.13023 |
| *FH* | c.914T>C (p.Phe305Ser) | SNV | 0.49616 | 0.17738 |

**Supplementary Table S5. Pathogenic/likely pathogenic germline variants not detected by tumor-only testing by technical lack of detection or by a VAF below the recommended thresholds according to tumor type and biological process.**

| **Cancer type** | **Total** | **Not detected** | | | | **Annual cases in U.S. (#)** | | | | **Annual cases worldwide (#)** | | | |
| --- | --- | --- | --- | --- | --- | --- | --- | --- | --- | --- | --- | --- | --- |
|  |  | **Number** | **%** | **Lower 95 CI** | **Upper 95 CI** | **Total number** | **Not detected (projection)** | | | **Total number** | **Not detected (projection)** | | |
|  |  |  |  |  |  |  | **Number** | **Lower 95 CI** | **Upper 95 CI** |  | **Number** | **Lower 95 CI** | **Upper 95 CI** |
| Breast cancer | 2,353 | 22 | 0.9% | 0.6% | 1.4% | 325,010 | 3,039 | 1,983 | 4,583 | 2,090,000 | 19,541 | 12,749 | 29,469 |
| Breast cancer (HRD or DDR) | 167 | 22 | 13.2% | 8.9% | 19.1% | - | - | - | - | - | - | - | - |
| Ovarian cancer | 1,070 | 7 | 0.7% | 0.3% | 1.3% | 21,410 | 140 | 66 | 287 | 300,000 | 1,963 | 930 | 4,020 |
| Ovarian cancer (HRD + DDR) | 124 | 7 | 5.6% | 2.8% | 11.2% | - | - | - | - | - | - | - | - |
| Prostate cancer | 2,209 | 8 | 0.4% | 0.2% | 0.7% | 191,930 | 695 | 345 | 1,363 | 1,300,000 | 4,708 | 2,340 | 9,230 |
| Prostate cancer (HRD + DDR) | 118 | 8 | 6.8% | 3.5% | 12.8% | - | - | - | - | - | - | - | - |
| Pancreatic cancer | 1,943 | 10 | 0.5% | 0.3% | 0.9% | 60,430 | 311 | 169 | 568 | 458,918 | 2,362 | 1,285 | 4,314 |
| Pancreatic cancer (HRD + DDR) | 123 | 10 | 8.1% | 4.5% | 14.3% | - | - | - | - | - | - | - | - |
| Colorectal cancer | 2,407 | 16 | 0.7% | 0.4% | 1.1% | 149,500 | 994 | 613 | 1,600 | 1,900,000 | 12,630 | 7,790 | 20,330 |
| Colorectal cancer (MMR) | 85 | 12 | 14.1% | 8.3% | 23.1% | - | - | - | - | - | - | - | - |
| Endometrial cancer | 1,506 | 4 | 0.3% | 0.1% | 0.7% | 66,570 | 177 | 73 | 459 | 382,069 | 1,015 | 420 | 2,636 |
| Endometrial cancer (MMR) | 31 | 3 | 9.7% | 3.4% | 24.9% | - | - | - | - | - | - | - | - |

**Supplementary Figure S3: Pre-test probability of pathogenic germline variant carrier status for 108 cancer patients with variants not detected by tumor-only sequencing as calculated using the BOADICEA V5 (HDR/DDR) or PREMM5 (Lynch syndrome) models.**


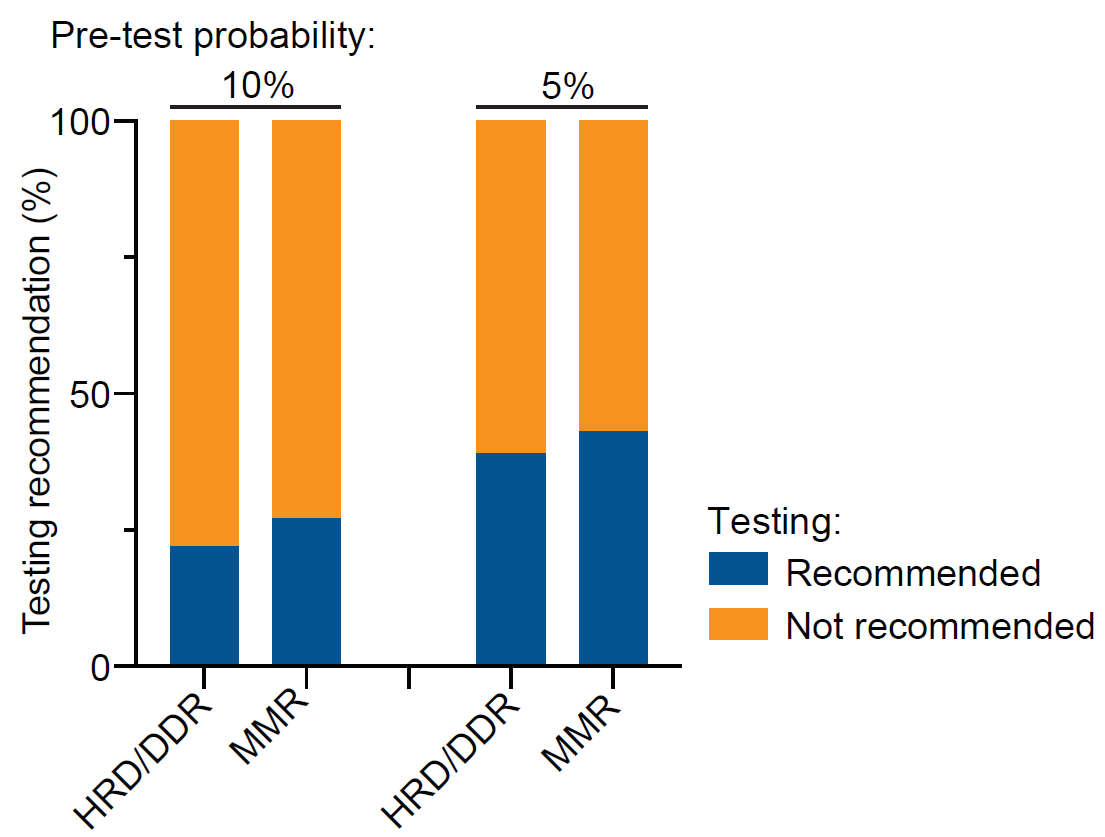

Supplement: supplementary material [file NIHMS1812141-supplement-supplementary_material.docx]
